# 4037 Assessing Leadership Skills in Translational Science Training: The Rockefeller University Leadership Survey

**DOI:** 10.1017/cts.2020.353

**Published:** 2020-07-29

**Authors:** Roger Vaughan, Michelle Romanick, Donna Brassil, Rhonda G Kost, Sarah Schlesinger, Barry S. Coller

**Affiliations:** 1 Rockefeller University

## Abstract

OBJECTIVES/GOALS: There is universal recognition of the importance of team science and team leadership. We have developed a semi-quantitative translational science specific team leadership competency assessment tool and have begun implementation studies to assess the impact of personalized feedback on the team science leadership skills of KL2 Clinical Scholars. METHODS/STUDY POPULATION: To create the instrument, we employed a modified Delphi approach by conducting a thorough literature review on Leadership to concretize the relevant constructs, then used these extracted constructs as a springboard for the Rockefeller Team Science Educators (TSE’s) to discuss and refine the leadership domain areas, collectively create domain-specific survey items. Further discussion helped refined the number, grouping, and wording. Scholars also contributed feedback in item development. We piloted the Leadership Survey by having all of the Rockefeller TSEs rate Clinical Scholars, and having each Scholar rate themselves. Each item was answered using a six-point Likert scale where a low score indicated poor expression and a high score represented excellent expression of the specific leadership attribute. RESULTS/ANTICIPATED RESULTS: Incorporation into a REDCap data base made consenting and rating process by TSE’s and the Scholars straightforward. The a priori domains (Foundational Leadership Competencies, Professionalism, Team Building and Team Sustainability, Appropriate Resource Use and Study Execution, and Regulatory Accountability) had high internal validity and good internal factor structure. The congruence between TSE and Scholar self-ratings were uniformly high, and discordance was often a function of “confidence” and “modesty” on the part of the scholar, rather than deficiency. Supporting comments were informative about performance barriers and mechanisms for improvement. Return of results allowed for the exploration of training gaps. Scholars were surveyed to gauge their reaction to the formal feedback. DISCUSSION/SIGNIFICANCE OF IMPACT: This quantification of team science leadership constructs has allowed for A)- the articulation of constructs essential for successful Translational Scientists to acquire during their training, B)- identification of gaps in that training and skill set, and C)- mechanisms for bolstering any identified gaps in these essential leadership constructs. CONFLICT OF INTEREST DESCRIPTION: None

